# Novel CRISPR-Cas9 iPSC knockouts for *PCCA* and *PCCB* genes: advancing propionic acidemia research

**DOI:** 10.1007/s13577-025-01193-z

**Published:** 2025-03-05

**Authors:** Emilio M. García-Tenorio, Mar Álvarez, Mónica Gallego-Bonhomme, Lourdes R. Desviat, Eva Richard

**Affiliations:** 1 Centro de Biología Molecular Severo Ochoa UAM-CSIC, Universidad Autónoma de Madrid, 28049 Madrid, Spain; 2 Instituto Universitario de Biología Molecular, Universidad Autónoma de Madrid, Madrid, Spain; 3 Centro de Investigación Biomédica en Red de Enfermedades Raras (CIBERER), ISCIII, Madrid, Spain; 4 Instituto de Investigación Sanitaria Hospital La Paz (IdiPaz), Madrid, Spain; 5 Madrid, Spain

**Keywords:** IPSC, Disease modelling, Propionic acidemia, Isogenic controls, CRISPR-Cas9

## Abstract

Propionic acidemia (PA) is a rare autosomal recessive metabolic disorder caused by mutations in the *PCCA* and *PCCB* genes, which encode subunits of the mitochondrial enzyme propionyl-CoA carboxylase (PCC). This enzyme deficiency leads to the accumulation of toxic metabolites, resulting in severe metabolic dysfunction. To create ideal in vitro disease models of PA with isogenic controls and provide a robust platform for therapeutic research, we generated two induced pluripotent stem cell (iPSC) lines with knockout (KO) mutations in the *PCCA* and *PCCB* genes using CRISPR-Cas9 gene editing in a healthy control iPSC line. The KO iPS cells were successfully established and characterized, confirming the presence of frameshift insertions and deletions in each target gene, as well as the loss of the corresponding transcript, protein expression, and activity. Additionally, the generated iPSC lines exhibit hallmark characteristics of pluripotency, including the potential to differentiate into all three germ layers. Our *PCCA* and *PCCB* KO iPSC models provide a valuable tool for studying the molecular mechanisms underlying PA and hold potential for advancing new therapeutic approaches.

**Supplementary Information:**

The online version contains supplementary material available at 10.1007/s13577-025-01193-z.

## Introduction

Propionic acidemia (PA; MIM#606054) is a rare autosomal recessive metabolic disorder caused by mutations in the *PCCA* and *PCCB* genes. These genes encode α (PCCA) and β (PCCB) subunits of the enzyme propionyl-CoA carboxylase (PCC, E.C.6.4.1.3), which is crucial for the catabolism of certain amino acids and lipids. Mutations in these genes lead to a deficiency in enzyme activity, resulting in the accumulation of toxic metabolites that can cause severe and life-threatening symptoms, including metabolic acidosis, developmental delay and organ dysfunction [1]. In PA, both the heart and brain can be significantly affected being the principal causes of morbidity and mortality [2]. The most common cardiac complications are QT prolongation, hypertrophy and dilated cardiomyopathy, which can lead to fatal cardiac arrhythmias [3]. Furthermore, patients with PA exhibit a wide range of neurological manifestations, including cognitive impairment, developmental delay and intellectual disability, as well as structural defects, and disorders of neuro-psychomotor development [4]. Early diagnosis and management are crucial to mitigating the damage to these vital organs in individuals with PA. Current therapeutic options are limited, underscoring the need for advanced disease models to better understand PA and explore potential treatments.

Induced pluripotent stem cells (iPSCs) offer a promising platform for modelling genetic diseases, as they can be derived from patient cells and have the ability to differentiate into various cell types [5, 6]. Patient-derived iPSCs may be compared with iPSC lines from healthy individuals used as controls. However, differences in phenotype might result from variations between cell lines rather than from the disease-causing variants themselves. In recent years, CRISPR-Cas9-mediated gene-editing technology has been effectively used for gene modification in iPSCs to generate models of inherited diseases [7]. The creation of isogenic pairs of disease-specific and control iPSCs, differing solely at the disease-causing mutation, has been employed to minimize variability, allowing the identification of disease-relevant differences in monogenic disorders [8]. In addition, parallel differentiation of these isogenic cell lines into relevant cell types facilitates detailed phenotypic analysis of cellular pathologies specific to the disease [7].

In this study, we utilized CRISPR-Cas9 to generate two knockout (KO) iPSC lines for the *PCCA* and *PCCB* genes from a healthy control iPSC line. These genetically modified iPSCs provide a valuable isogenic system to study the molecular and pathophysiologic mechanisms of PA and represent a versatile tool for investigating potential therapeutic interventions in this rare disease.

## Materials and methods

### Cell line and culture

Human iPSC control line SCTi003-A (StemCell™ Technologies #200–0511, Vancouver, BC, Canada), a hiPSC line derived from peripheral blood mononuclear cells of a healthy female donor, was used in this work. iPS cells were cultured in 60 mm tissue culture dishes coated with Matrigel (hESC-qualified matrix, Corning, New York, NY, USA), using mTESR™ Plus medium (StemCell™ Technologies) at 37 °C with 5% CO_2_ and 20% O_2_, with medium changes every other day. The iPSCs were passaged every four days using ReleSR™ (StemCell™ Technologies) or StemPro Accutase (Gibco, Waltham, MA, USA) and 10 µM Rock inhibitor (StemCell™ Technologies) at a 1:3–1:5 splitting ratio.

### CRISPR-Cas9-mediated gene KO

The online tools available at https://bioinfogp.cnb.csic.es/tools/breakingcas/ and http://crispor.gi.ucsc.edu/ were used to design guides with high specificity for *PCCA* and *PCCB* target sites while minimizing predicted off-targets located in genomic regions (Table 1). On the day of nucleofection, 2 × 10^5^ cells (80% confluency and passage 4) were nucleofected using a Neon Electroporation Kit (Invitrogen, #MPK1025, Waltham, MA, USA) and the Neon Transfection System (Invitrogen), according to provider´s instructions. Ribonucleoprotein (RNP) complexes were formed by the combination of 45 pmol of Cas9 (*Streptococcus pyogenes*, Integrated DNA Technologies (IDT), Newark, NJ, USA) and 55 pmol of RNA duplex constituted by tracrRNA and RNA guide (IDT); and with 55 pmol of enhancer (IDT). Cells were transferred into a single well of a Matrigel-coated 12-well plate and cultured in mTESR™ Plus medium. Several days after nucleofection, the cells were transferred to Matrigel-coated 60 mm dishes in mTESR™ Plus medium. Cells were then sorted using a FACSAria Fusion (BD Biosciences, Franklin Lakes, NJ, USA) into Matrigel-coated 96-well plates with mTESR™ Plus medium supplemented with 1X CloneR (StemCell™ Technologies) for clonal expansion.

**Table 1:** Primers and RNA guides used in the study

	Target	Oligonucleotides (5′–3′)*
Target sequences (gRNA)	*PCCA* exon 5	gRNA: UUCAUUCAGACUCACAGCTT
	*PCCB* exon 5	gRNA: AUGGACCAGGCCAUAACGGU
Target knockout analysis	*PCCA* exon 5	Fwd: GTCTCCTCAGGTTCATGTGA
		Rev: AGTAGATAGATTCATTCAGACTCACAG
	*PCCB* exon 5	Fwd: CAGATCATGGACCAGGCCATAAC
		Rev: GAGGAGAGTTGGGAGAAGCTCTGGC
Sanger sequencing	*PCCA* exon 5	Fwd: GTCTCCTCAGGTTCATGTGA
		Rev: AGTAGCGAATTCGACCACAT
	*PCCB* exon 5	Fwd: GGGAGTGAAACCAGTGTTACTGCC
		Rev: GAGGAGAGTTGGAGAAGCTCTGGC
Next generation sequencing	*PCCA* exon 5	Fwd: ACACTGACGACATGGTTCTACAGTCTCCTCAGGTTCATGTGA
		Rev: TACGGTAGCAGAGACTTGGTCTAGTAGCGAATTCGACCACAT
	*PCCB* exon 5	Fwd: ACACTGACGACATGGTTCTACAGGGAGTGAAACCAGTGTTACTGCC
		Rev: TACGGTAGCAGAGACTTGGTCTGAGGAGAGTTGGAGAAGCTCTGGC
RT-PCR	*PCCA* exon 2–7	Fwd: GTGTCCCGTAATCTTGGTTC
		Rev: CAGGGATTGTATTAACCTCTGCTT
Quantitative PCR	*PCCA* exon 1–4	Fwd: GGACCCTGAAGCATGTTCTGTAC
		Rev: TAACCCGACATGCAATTTCTCC
	*PCCB* exon 1–3	Fwd: GCACAAGCGAGGAAAGCTAAC
		Rev: CGCTGTCTCCAGGAAACTTATTC
Off-target analysis *PCCA*	*LINC02869*	Fwd: GCAAATCCGCAACATCAGC
		Rev: AGTGAGGAGGCACAGCAAC
	*STX11*	Fwd: ACCAAGACCCCATCTTCTGTG
		Rev: CATTTCACCCACTGCCTGG
	ENSG00000250971	Fwd: GGACTGAGACTGAACCCTTGG
		Rev: CCACCCTGAGGCACACTAAAG
	AC064870.5	Fwd: GGCTGAGTCCCTGTGAAGAAG
		Rev: CTTATTCATGGTCCCACAGCAG
Off-target analysis *PCCB*	*SLC7A14-AS1*	Fwd: AAAGCAGTGAGCCAGTGGG
		Rev: TTCCCTGTGAAAGCGGTGG
	AC117526.2	Fwd: AGGTGGAAGTTTGCTGAGTGG
		Rev: AATGCTCTTACTGGCTGATGC
	AL034431.16	Fwd: ACTCTTGCTCTCAGCCCATTG
		Rev: CACCTCAGACTACTCTGGCAC
	*SLC2A13*	Fwd: CCTGACCTCAAGTATCCACCC
		Rev: GTGGCTGTTCCAAACAAGGTC
Mycoplasma detection	Mycoplasma species	Forward primers:
		CGCCTGAGTAGTACGTTCGC
		CGCCTGAGTAGTACGTACGC
		TGCCTGGGTAGTACATTCGC
		TGCCTGAGTAGTACATTCGC
		CGCCTGAGTAGTATGCTCGC
		CACCTGAGTAGTATGCTCGC
		CGCCTGGGTAGTACATTCGC
		Reverse primers:
		GCGGTGTGTACAAGACCCGA
		GCGGTGTGTACAAAACCCGA
		GCGGTGTGTACAAACCCCGA

^*^gRNA RNA guide, *Fwd* Forward, *Rev* Reverse

### PCC enzymatic assay

PCC activity was assayed by measuring the enzyme-dependent incorporation of radiolabelled bicarbonate into non-volatile products, as previously described [9].

### Karyotype analysis

Cells were exposed to 10 μg/ml Colcemid® Solution (Irvine Scientific, Santa Ana, CA, USA) for 3 h at 37 °C, then dissociated using accutase, treated with a hypotonic solution, and fixed with Carnoy’s fixative in preparation for karyotype analysis. A minimum of 20 metaphase cells were analyzed. Karyotype analysis was performed by the Molecular Cytogenetics and Genome Editing Unit from Centro Nacional de Investigaciones Oncológicas (CNIO, Madrid, Spain).

### DNA extraction and PCR mismatch

Genomic DNA was extracted from iPSCs using QIAamp DNA Mini Kit (Qiagen, Venlo, The Netherlands) following the manufacturer´s recommendations. Editing was verified by PCR mismatch using 100 ng of DNA from the cell population, the specific primers for target knockout analysis (Table 1), and the Supreme NZYTaq II 2 × Green Master Mix (NZYTech, Lisbon, Portugal).

### Sequencing

PCR products were cloned in pGEM®-T Easy Vector System I (Promega Corporation, Madison, WI, USA). Plasmid DNA was purified using NZYMiniprep Kit (NZYtech) and was subjected to Sanger sequencing (Macrogen, Seoul, South Korea). At least 20 colonies were sequenced. Deep sequencing was performed using amplicons generated in a first PCR with fusion primers containing a specific sequence plus a common tag (Table 1). Libraries preparation and sequencing were carried out at Fundación Parque Científico de Madrid (Campus Cantoblanco, Madrid, Spain) under protocols developed and optimized for next-generation amplicon sequencing. For off-target analysis, PCR products were purified using E.Z.N.A Cycle Pure kit (Omega Bio-Tek, Norcross, GA, USA) before Sanger sequencing.

### RNA extraction, RT-PCR and RT-qPCR

To assess the splicing effects in the *PCCA* KO cell line, total RNA was isolated using TRIzol® Reagent (Life Technologies, Waltham, MA, USA), and 1 μg of RNA was retrotranscribed using NZY First-Strand cDNA Synthesis kit (NZY Tech). RNA from the unedited iPSC line was used as a control. PCR was performed using the primers hybridizing to exons 2 and 7 of the *PCCA* gene (Table 1), and FastStart Taq DNA polymerase (Roche Diagnostics GmbH, Mannheim, Germany). PCR products corresponding to transcripts were separated on an agarose gel and purified using a QIAquick gel extraction kit (Qiagen) before Sanger sequencing.

To assess the effect of gene KO on their respective target mRNA levels, cDNA was obtained by retrotranscription of 500 ng of total RNA as described above. *PCCA* and *PCCB* were then amplified with specific primers designed upstream of the edition site (Table 1), using PerfeCTa SYBR Green FastMix (Quanta Biosciences, Beverly, MA, USA) in a CFX Opus 384 real-time PCR system (Bio-Rad, Hercules, CA, USA) following the manufacturer’s instructions. Data were analysed using Bio-Rad CFX Maestro software, and relative quantification to the wild-type iPSC line was performed according to 2^−∆∆CT^ method, using *GAPDH* as an endogenous control.

### Immunoblot analysis

Protein extracts were prepared from frozen cell pellets through a freeze–thaw lysis process using a buffer containing Tris–HCl pH 7.4, 10% glycerol, 150 mM NaCl, 0.1% Triton X-100, and protease and phosphatase inhibitor cocktail (Sigma-Aldrich, St. Louis, MO, USA). Lysates were then centrifuged for 10 min at 4 °C, after which the supernatant was collected, and protein concentration was measured using the Bradford method (Bio-Rad). 40 µg of protein from each sample were loaded onto 4–12% NuPAGE™ Precast Gels (Invitrogen). After electrophoresis, proteins were transferred to a nitrocellulose membrane in an iBlot Gel transfer device (Invitrogen), then incubated with the primary antibodies at 4 °C overnight (Table 2), and subsequently incubated with the secondary antibodies at room temperature for 1 h (Table 2). GAPDH was used as a loading control. Enhanced chemiluminescence reagent (ECL, GE Healthcare, Chicago, IL, USA) and SuperSignal™ West Femto Maximum Sensitivity Substrate (Thermo Fisher Scientific, Waltham, MA, USA) were used for protein detection.

**Table 2:** Antibodies used in the study

	Antibody	Dilution	Company Cat# and RRID
For immunocytochemistry/flow cytometry			
Pluripotency markers	Mouse IgG anti-Oct4	1:60	Santa Cruz Cat# sc-5279, AB_628051
	Rabbit IgG anti-Sox2	1:100	Fisher Thermo Scientific Cat# PA1-16968, AB_2195781
	Goat IgG anti-Nanog	1:25	R&D Cat# AF1997, AB_355097
	Rat IgM anti-SSEA3	1:3	Hybridoma Bank Cat# MC-631, AB_528476
	Mouse IgG anti-SSEA4	1:3	Hybridoma Bank Cat# MC-813-70, AB_528477
	Mouse IgM anti-Tra1-60	1:200	Millipore Cat# MAB4360, AB_2119183
	Mouse IgM anti-Tra1-81	1:200	Millipore Cat# MAB4381, AB_177638
Differentiation markers	Rabbit IgG anti-α−1-fetoprotein	1:400	Dako Cat# A0008, AB_2650473
	Mouse IgG anti-α-smooth muscle actin	1:400	Sigma-Aldrich Cat# A5228, AB_262054
	Mouse IgG anti-β-III-Tubulin Tuj1	1:500	Covance Cat# MMS-435P, AB_231377
Secondary antibodies	Alexa 555 Donkey anti-mouse IgG	1:200	Thermo Fischer Cat# A-31570, AB_2536180
	Alexa 488 Donkey anti-Rabbit IgG	1:200	Thermo Fischer Cat# A-31572, AB_162543
	Alexa 647 Donkey anti-Goat IgG	1:200	Thermo Fischer Cat# A-21447, AB_2535864
	Alexa 488 Goat anti-Rat IgM	1:200	Thermo Fischer Cat# A-21212, AB_2535798
	Alexa 647 Goat anti-Mouse IgM	1:200	Thermo Fischer Cat# A-21238, AB_2535807
	Cy3 Donkey anti-Mouse IgM	1:200	Jackson Cat# 715-165-140, AB_2340812
	Alexa 647 Goat anti-mouse IgG	1:600	Thermo Fischer Cat# A-21235, AB_2535804
	APC Rat anti-mouse APC	1:150	BD Pharmingen Cat# 550676, AB_398464
For Western Blot	Mouse IgG anti-PCCA	1:500	Santa Cruz Cat# sc-374341, AB_10987856
	Mouse IgG anti-PCCB	1:500	Santa Cruz Cat# sc-393929
	Mouse IgG anti-GAPDH	1:5000	Abcam Cat# ab8245, AB_2107448
	Goat anti-Mouse	1:2000	Santa Cruz Biotechnology Cat# sc-2031, AB_631737

### Immunostaining

iPSCs were seeded onto matrigel-coated 15 μ-Slide 8 well culture plates (Ibidi, GmbH, Gräfelfing, Germany), fixed with Formaline Solution 10% (Sigma-Aldrich), and stained with anti-OCT4/NANOG/SOX2/SSEA-3/SSEA-4/TRA-1–60 and TRA-1–81 at 4 °C overnight using the dilutions previously described [10]. Tris-buffered saline was used for washing, blocking and antibody incubation solutions supplemented with Triton-X100 (Sigma-Aldrich) (Nuclear markers: 0.1% for washes and 0.3% for blocking and antibody incubation solutions) and Tween-20 (Merck Millipore, Burlington, MA, USA) (Surface markers: 0.2% for washes and 0.4% for blocking and antibody incubation solutions). Blocking and antibody incubation solutions were also supplemented with 3% Donkey Serum (Sigma-Aldrich). After several washes, cells were labelled with secondary antibodies for 2 h at 37 °C, followed by 30 min at room temperature. For nucleus staining DAPI (Invitrogen, 1:10,000) was used. Images were obtained using a Nikon A1R fluorescence microscope. Antibodies details are in Table 2.

### Flow cytometry analysis

The pluripotency markers SSEA-4, TRA-1–60, and TRA-1–81 were also examined by flow cytometry, as detailed in [10], using a BD FACSCanto™ A instrument (BD Biosciences), FACSDiva and FlowJo v10.10 software program. Unstained iPSCs and matching isotype antibodies served as negative controls to eliminate non-specific fluorescence data.

### In vitro three-germ-layer differentiation assay

Embryoid body (EB) formation was applied by plating the dissociated cells onto 96-well v-bottom, low attachment plates (Deltalab, Barcelona, Spain). Emerging EB were replated on matrigel-coated 15 µ-Slide 8 well culture plates (Ibidi) for another 18 days with different mediums as described [10]. Cells were fixed with Formaline Solution 10% and stained with endodermal (α−1-Fetoprotein, AFP), mesodermal (α-Smooth muscle actin, SMA) and ectodermal (β-III-Tubulin Tuj1, TUJ1) differentiation markers (Table 2).

### Short tandem repeat (STR) analysis

DNA fingerprinting analysis was conducted using the AmpFLSTR® Identifiler® PCR Amplification Kit (Thermo Fisher Scientific). For this process, 1 ng of DNA was used to evaluate highly polymorphic regions containing short tandem repeats by amplifying the following markers: D7S820, CSF1P0, Th01, D13S317, D16S539, vWA, TPOX, D5S818, and Amelogenin for determining sex. Samples were analyzed on a 3730 DNA Analyzer (Applied Biosystems, Foster City, CA, USA), and data processing was done using GeneMapper® v4.0 software at the Parque Científico de Madrid, Campus Moncloa, UCM, Madrid, Spain.

### Mycoplasma detection

Mycoplasma test was performed using the PCR method [11]. A positive sample with mycoplasma was used as a control.

## Results

### Generation of *PCCA* and *PCCB* KO iPSC lines

**Fig. 1:**
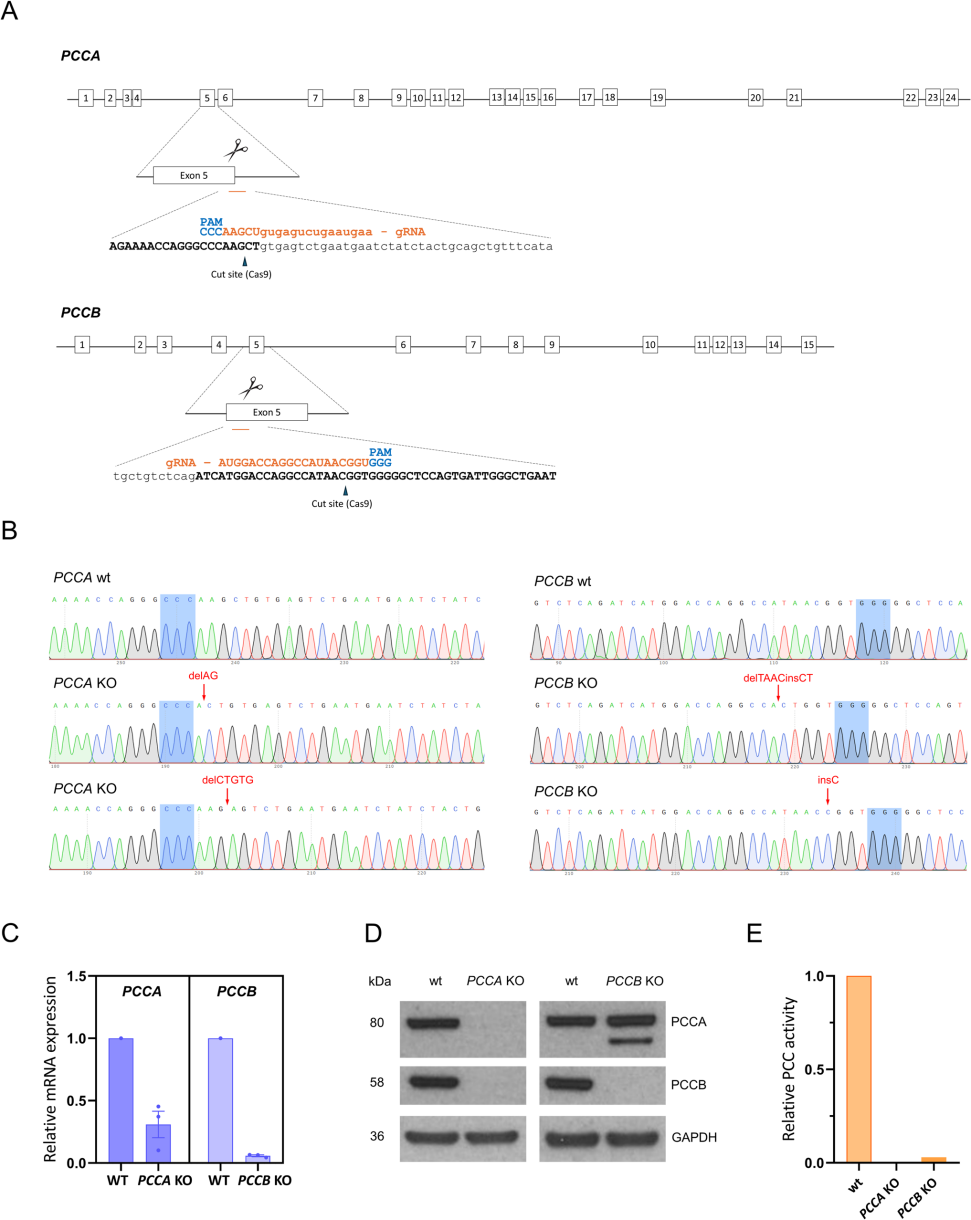
Generation of the *PCCA* and *PCCB* KO iPSC lines. **A** Schematic representation of *PCCA* and *PCCB* genes. The specific RNA guides are indicated in both genes (orange) with their respective PAM sequences (blue). **B** Sanger sequencing results after subcloning both alleles of the generated KO iPSC lines. PAM sequences are highlighted in blue. **C** RT-qPCR for *PCCA* and *PCCB* genes in their respective KO iPSC lines compared to the wild-type (wt). The relative quantification data presented include the mean and standard error of mean for three biological replicates. **D** Western Blot against PCCA and PCCB proteins in the wild-type iPSC line (wt) and KO iPSC lines. GAPDH was used as a loading control.** E** Histogram representation of PCC activity in the wild-type iPSC line (wt) and KO iPSC lines

To generate KO iPSC lines for the *PCCA* and *PCCB* genes, we designed specific guides targeting key regions within each gene. For both cases, the guides were directed at exon 5, an essential region for the proper function of the encoded proteins (Fig. 1A). The guides were selected based on their predicted on-target efficiency and low off-target potential, using the CRISPOR and Breaking-Cas design tools. After nucleofection with CRISPR-Cas9 and sgRNA RNP complexes, up to 12 iPSC colonies were manually picked and screened for insertion-deletions (indels) within the target regions using PCR mismatch, revealing the presence of edition in all of them. Subsequently, cell sorting and expansion of individual clones was performed to ensure the isolation of pure homogeneous clones. The mutations in the selected clones were identified by Sanger sequencing after subcloning to separate the two alleles (Fig. 1B), and the results were further confirmed by deep sequencing of each region (data not shown). Our sequencing results revealed the presence of biallelic frameshift mutations within the target exons of both *PCCA* and *PCCB* genes. Specifically, in the *PCCA* KO iPSC line, we identified two frameshift-causing mutations: 2-bp (c.411_412del) and 5-bp (c.413_414 + 3del) deletions. The c.411_412del variant introduces a premature stop codon 30 amino acids downstream of the edited site (p.(Gln137Hisfs*30)), while c.413_414 + 3del disrupts the 5′ splice site of exon 5, predictably causing a splicing defect. This splicing alteration was confirmed by RT-PCR and sequencing of the transcript, which revealed the inclusion of 40 bp of the intronic sequence following the deletion (r. 413_414delins40), due to the activation of a cryptic splice site. This alteration was also predicted to result in a premature stop codon (p.(Ala138Glufs*4)). From the edited *PCCB* iPSC clones, we selected a compound heterozygous clone in which one allele carries a 1 bp insertion (c.448_449insC) and the other allele a 4 bp deletion with a 2 bp insertion (c.446_449delinsCT), both resulting in frameshifts and premature stop codons at residues 10 (p.(Val151Glyfs*10)) and 11 (p.(Ile149Thrfs*11)) downstream of the edition, respectively.

These mutations in both genes were predicted to be disease-causing by Mutation Taster (http://www.mutationtaster.org/). Specifically, the mutations lead to the absence of part of the biotin carboxylase domain in the *PCCA* gene, and of the carboxyl transferase domain in the *PCCB* gene, respectively. Our results showed a reduction in *PCCA* and *PCCB* mRNA levels in the respective KO iPSCs (Fig. 1C), which may be due to nonsense-mediated mRNA decay, triggered by the premature stop codons generated by the editing process. If any transcripts are produced, an unstable truncated protein would be generated and rapidly degraded, as confirmed by Western Blot analysis and PCC activity measurement. These assays revealed a complete absence of protein expression (Fig. 1D) and enzymatic activity (Fig. 1E) in both *PCCA* and *PCCB* KO iPSC lines, confirming the successful generation of the KO lines. In the *PCCA* KO iPSC line we observed a loss of both PCCA and PCCB protein expression, as expected. This occurs because, although the PCCB protein is expressed, it is unstable and prone to degradation in the absence of the PCCA subunit [12]. However, in the *PCCB* KO line, PCCA is still expressed in the absence of PCCB, although a smaller degradation product is also observed (Fig. 1D).

Additionally, Sanger sequencing analysis revealed the absence of off-target events in the top four predicted off-target sites for both clones (Fig. S1).

### Characterization of *PCCA* and *PCCB* KO iPSC lines

**Fig. 2:**
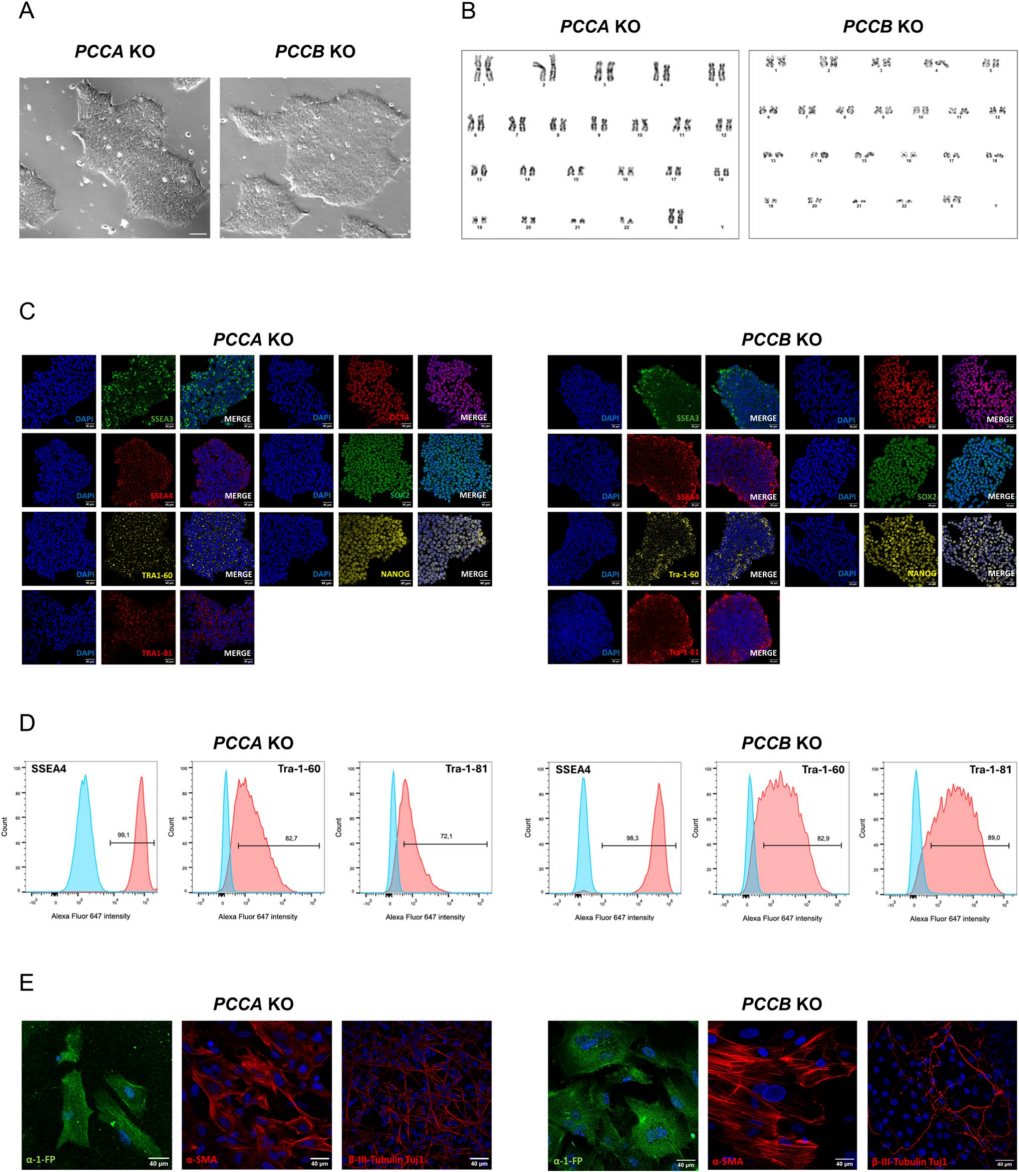
Characterization of the *PCCA* and *PCCB* KO iPSC lines. **A** Representative phase contrast images of iPSC colonies in both KO iPSC lines (scale bar 40 µm). **B** Representative G-banding karyotype images of normal 46, XX for both cell lines. **C** Immunofluorescence analysis for OCT4/NANOG/SOX2/SSEA-3/SSEA-4/TRA-1–60 and TRA-1–81 pluripotency markers (scale bar 40 µm). **D** Flow cytometry analysis for SSEA-4/TRA-1–60 and TRA-1–81 surface pluripotency markers. **E** In vitro differentiation analysis by immunofluorescence for AFP, SMA and TUJ1 proteins (scale bar 40 µm)

We performed a comprehensive characterization of the KO clones for the *PCCA* and *PCCB* genes to confirm their genomic integrity and evaluate their pluripotency potential (Fig. 2). Both KO iPSC lines exhibited typical stem cell-like morphology, forming colonies with tightly packed cells that had large nucleus-to-cytoplasm ratios and prominent nucleoli (Fig. 2A). Karyotyping was conducted to assess the chromosomal integrity of each clone and verify that no cytogenetic abnormalities were introduced during the gene editing process. The cell lines maintained a normal molecular karyotype (46, XX) (Fig. 2B).

Immunofluorescence staining demonstrated robust expression of pluripotency markers in both iPSC lines. Nuclear markers OCT4, SOX2 and NANOG, as well as surface markers SSEA-3, SSEA-4, TRA-1–60 and TRA-1–81 were all highly expressed (Fig. 2C). Flow cytometry analysis further confirmed that over 72% of cells expressed the surface markers SSEA-4, TRA-1–60 and TRA-1–81 across the cell populations (Fig. 2D).

Additionally, the functional pluripotency of the iPSCs was evaluated through directed differentiation into the three germ layers. This was achieved by using EB formation, followed by immunocytochemical analysis of lineage-specific markers: AFP for endoderm, SMA for mesoderm and TUJ1 for ectoderm (Fig. 2E). Positive expression of all three markers confirmed the strong differentiation potential of the *PCCA* and *PCCB* KO iPSC lines, indicating that the gene knockouts did not impair their ability to differentiate into the three germ layers.

Finally, STR profiling at 9 loci validated the cell identity of the *PCCA* and *PCCB* KO iPSCs by matching them to their parental wild-type cells (Fig. S2A). Rigorous mycoplasma testing confirmed that both cell lines were free of contamination, preserving the integrity of the experimental conditions and results (Fig. S2B).

## Discussion

Over the last decade, the generation of iPSCs has revolutionized biomedical research, enabling scientists to explore previously inaccessible aspects of human biology, such as early developmental stages, cellular differentiation, and the progression of genetic diseases at a cellular level. This technology has also paved the way for advancements in personalized medicine and regenerative therapies by allowing the creation of patient-specific cell lines [13].

Previously, we generated two iPSC lines from patients-derived fibroblasts with defects in the *PCCA* and *PCCB* genes [10, 14], as well as an isogenic *PCCB*-corrected cell line using CRISPR-Cas9 gene editing [15]. Working with patient-derived iPSCs and generating appropriate isogenic controls using CRISPR-Cas9 technology poses significant challenges, particularly in the case of PCCA-deficient patients in which large genomic deletions are frequent [16]. These PA iPSC lines initially provided an essential platform for dissecting the molecular mechanisms underlying the disease, especially in tissues highly affected by PA deficiency, such as the heart. In patient-derived iPSC cardiomyocytes, we observed upregulation of cardiac-enriched miRNAs and alterations in key signaling pathways, including increased cardiac damage markers and cardiac ion channels, oxidative stress, reduced mitochondrial respiration, impaired autophagy, and lipid accumulation [17, 18].

In this study, we focused on creating isogenic iPSC lines deficient in *PCCA* and *PCCB* genes, successfully generating two KO iPSC lines from a control line. The precision of the knockout strategy, demonstrated by the introduction of out-of-frame indels within exon 5, led to the generation of premature stop codons in both genes, resulting in a loss of the corresponding transcript, protein expression, and function, as confirmed by in silico predictions and experimental validation. The generated KO iPSC lines exhibit genomic integrity and maintain pluripotency characteristics. This not only highlights the effectiveness of CRISPR technology for creating disease-relevant models [19, 20] but also underscores its potential for investigating PA with isogenic disease models.

PA patients suffer irreversible cerebral complications due to the currently limited therapeutic options. Brain imaging studies in PA patients have revealed a range of abnormalities, including bilateral basal ganglia damage, generalized brain atrophy, delayed myelination, white matter changes, cerebral atrophy, intracranial and cerebellar hemorrhage, and medullary lesions [4]. The exact pathophysiological mechanism by which PA causes brain complications remains unclear [21], although several mechanisms involving the accumulation of propionate and its toxic metabolites have been proposed. These include disruptions in signaling pathways like cAMP and type I protein kinase, altered phosphorylation of the cytoskeletal protein, glial fibrillary acidic proteins and vimentins, inhibited lipid biosynthesis (potentially causing demyelination), impaired ganglioside production, oxidative stress, mitochondrial dysfunction, and altered gene expression in neuronal and glial cells [4, 22]. To our knowledge, relevant cellular models of PA for studying neurological alterations have not been described to date, with iPSCs representing a promising tool for their generation, as has been demonstrated for other neurometabolic disorders, such as tyrosine hydroxylase deficiency [23].

Moving forward, our next goal is to develop cerebral organoids from these KO iPSCs. Organoids provide a unique opportunity to mimic the 3D architecture and cellular diversity of the human brain, offering deeper insights into disease mechanisms [24]. By developing these cerebral organoids, we aim to advance our understanding of the neurological and biological complexities associated with PA and investigate potential differences between the *PCCA* and *PCCB*-affected cells, given that *PCCB* has been associated with various neurodevelopmental and neuropsychiatric disorders such as autism [25, 26] and schizophrenia [27], while the presence of autistic features in *PCCA* deficient patients is far less common [25, 26].

In conclusion, the use of the wild-type line alongside the *PCCA* and *PCCB* KO iPSC lines provides a robust platform for future research aimed at understanding the pathophysiology of PA disease. These models also represent a critical step toward developing novel therapeutic strategies, as they will be instrumental for investigating potential treatments, particularly in gene-specific contexts.

## Supplementary Information

Below is the link to the electronic supplementary material.

Supplementary file 1 (PDF 998 KB)

## Data Availability

Data will be made available on request.
