# Supplementary material for: Novel CRISPR-Cas9 iPSC knockouts for PCCA and PCCB genes: advancing propionic acidemia research
Source: Hum Cell. 2025 Mar 5;38(3):64. doi: 10.1007/s13577-025-01193-z (PMC11882705; doi:10.1007/s13577-025-01193-z)

Figure 1S: Evaluation of potential off-target sites by Sanger sequencing.
The off-target sites presented in this work were selected according to score predicted in Breaking-Cas. For each off-target site (OT), the genomic locus or contig is specified according to ENSEMBL database, and its sequence is shown with mismatches highlighted in red and PAM in blue. In all cases the sequence for the unedited iPSC line (control) was compared to the *PCCA* or *PCCB* KO line. The analysis revealed the absence of off-target events within the top four predicted off-target sites for both clones.

***PCCA* gRNA target**:TTCATTCAGACTCACAGCTTGGG

OT1: *LINC02869* (chr 1). TCCAGTCATACTCACAGCTTGGG

Control

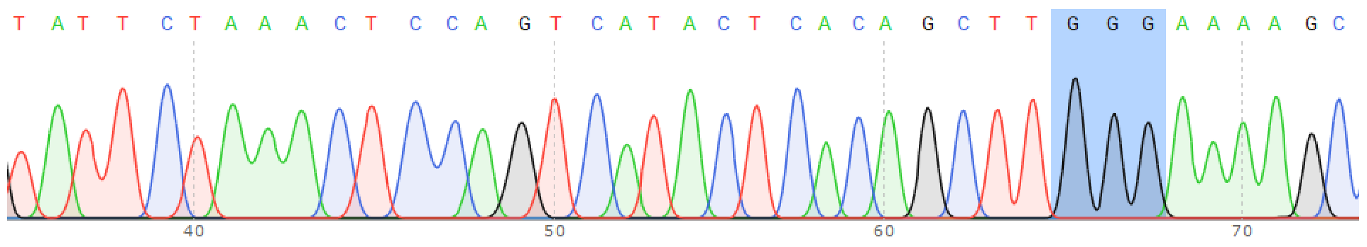

PCCA KO

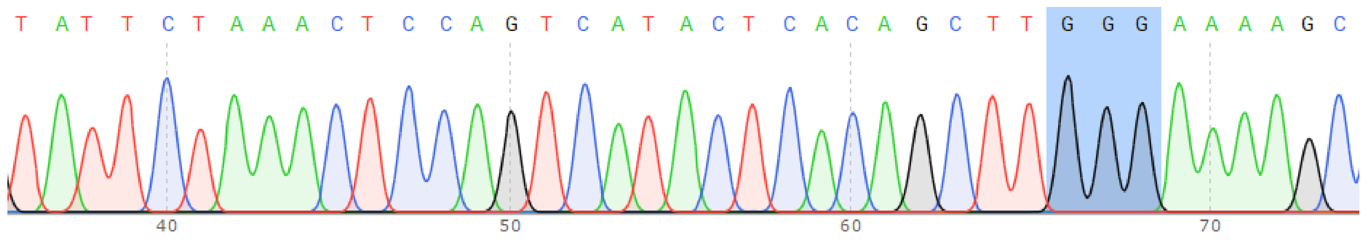

OT2: *STX11* (chr 6). CCCAAGCTGTGAGTCAGAAAGTC

Control

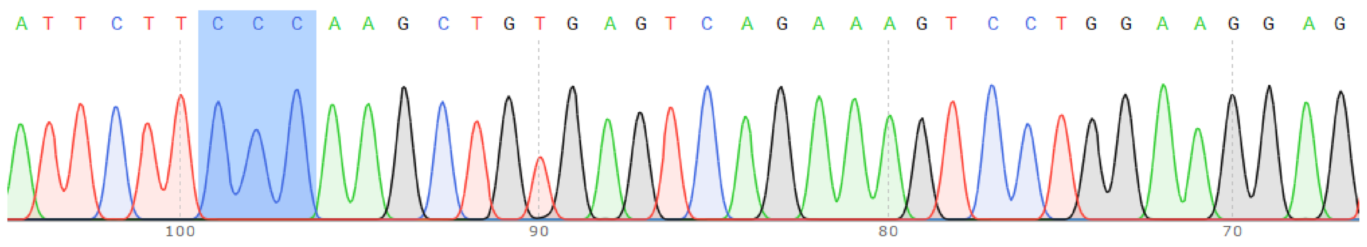

PCCA KO

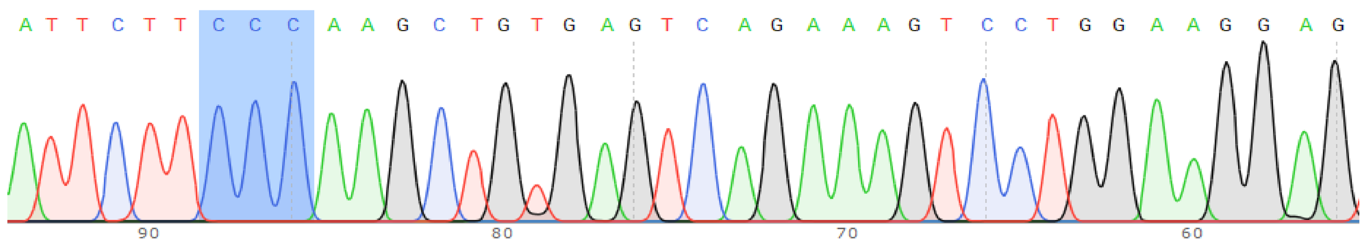

OT3: ENSG00000250971 (chr 4). TGAATTAGGACTCACAGCTTTGG

Control

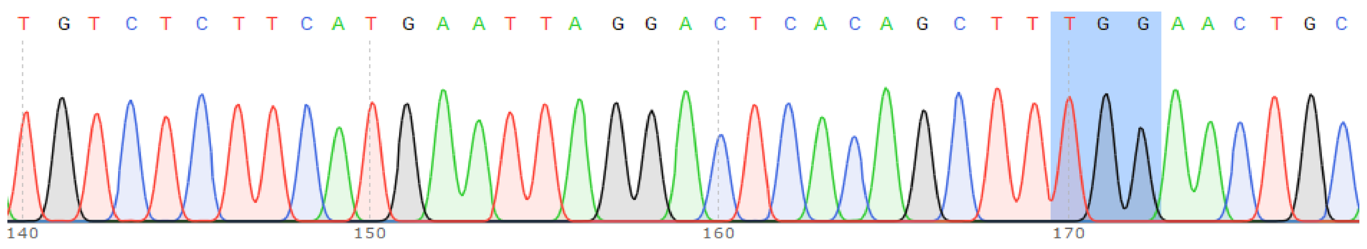

PCCA KO

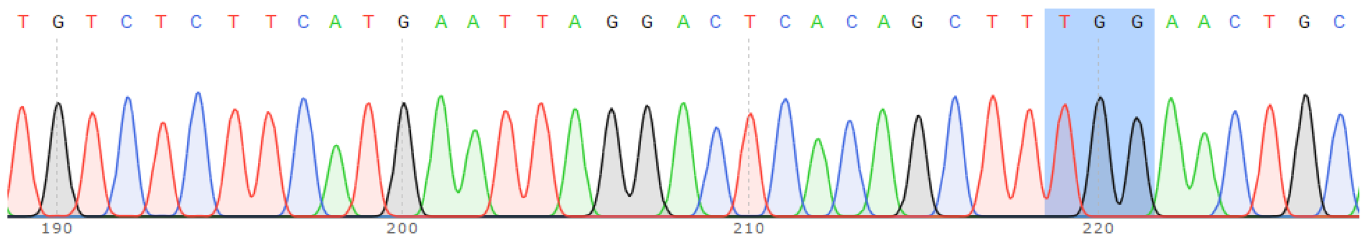

OT4: AC064870.5 (chr 2). CCCAAGCTGTGAGTGAGAATGGC

Control

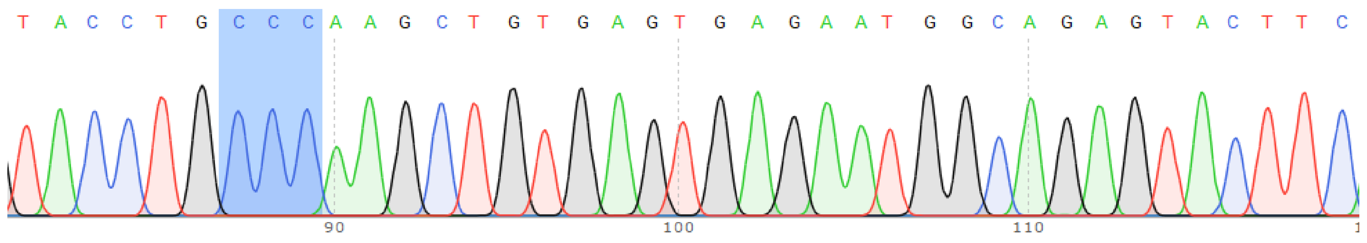

PCCA KO

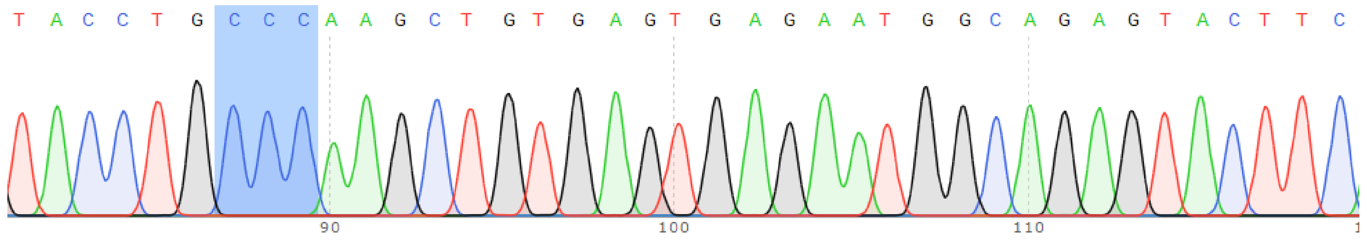

***PCCB* gRNA target**:ATGGACCAGGCCATAACGGTGGG

OT1: *SLC7A14-AS1* (chr 3). CCAACCTTTATGGACTGGTCTAG

Control

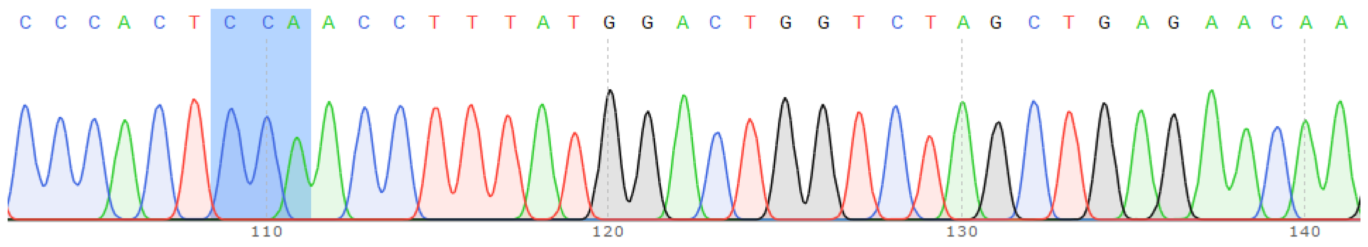

PCCB KO

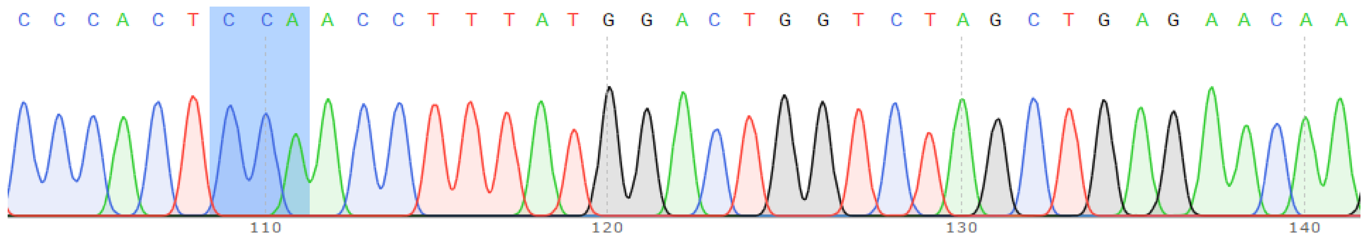

OT2: AC117526.2 (chr 5). CTGGAACAGCCCATAATGGTAGG

Control

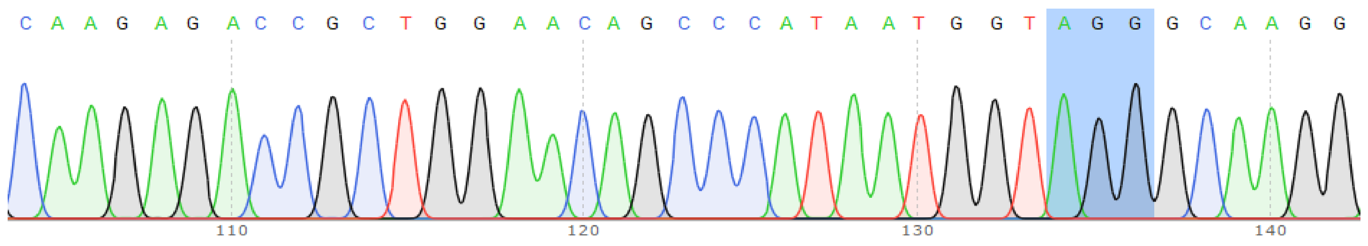

PCCB KO

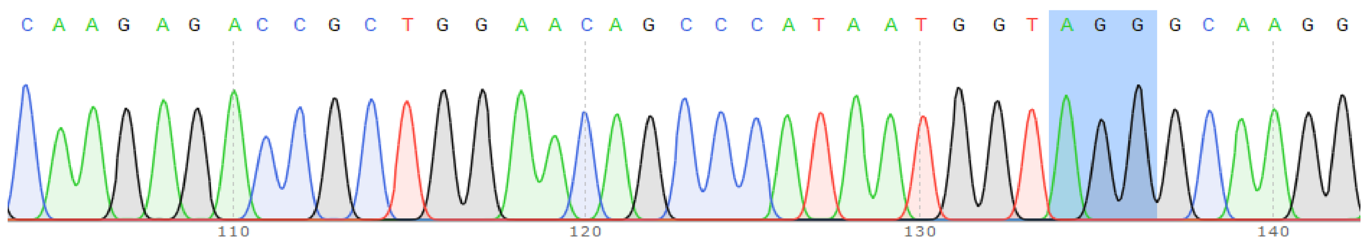

OT3: AL034431.16 (chr 20). ATGGAGTAGCCCATAAGGGTTGG

Control

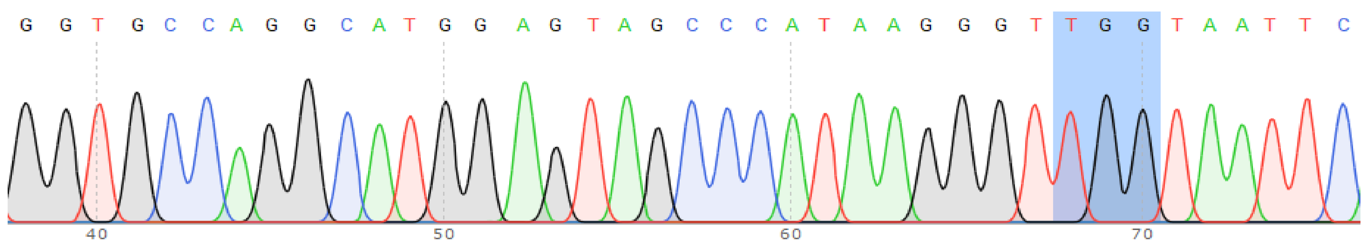

PCCB KO

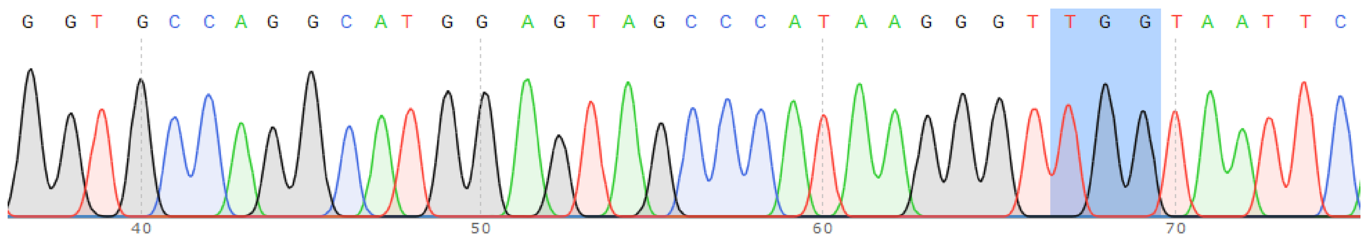

OT4: *SLC2A13* (chr 12). CCCACCTTTATCCCCTGGTTCAT

Control

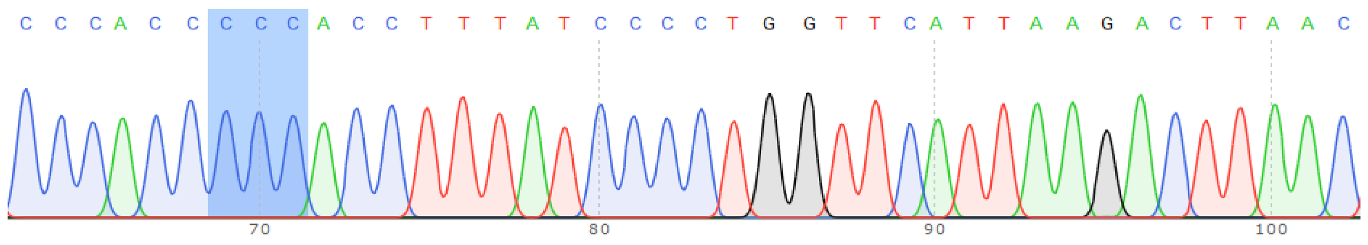

PCCB KO

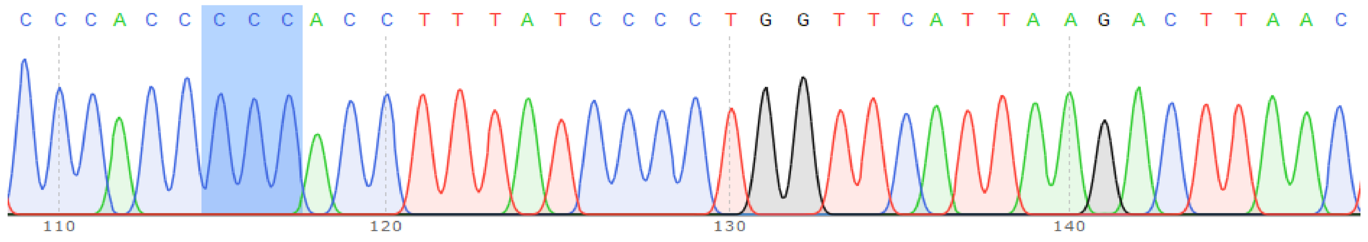

Figure 2S

(A) STR profiling guaranteed the genetic identity between the established iPSC lines and the parental line. (B) PCR showing negative mycoplasma test in the *PCCA* and *PCCB* KO iPSC lines. A contaminated cell line was used as a positive control (C+). M (DNA Ladder). B (blank).

A.

Marker	Parental wild-type iPSC line		*PCCA* KO iPSC line		*PCCB* KO iPSC line	
	Size (Alelle)		Size (Alelle)		Size (Alelle)	
**D7S820**	268 (9)	280 (12)	268 (9)	280 (12)	268 (9)	280 (12)
**CSF1PO**	320 (10)	320 (10)	320 (10)	320 (10)	320 (10)	320 (10)
**Th01**	187 (9.3)	187 (9.3)	187 (9.3)	187 (9.3)	187 (9.3)	187 (9.3)
**D13S317**	230 (11)	230 (11)	230 (11)	230 (11)	230 (11)	230 (11)
**D16S539**	277 (11)	277 (11)	277 (11)	277 (11)	277 (11)	277 (11)
**vWA**	180 (17)	180 (17)	180 (17)	180 (17)	180 (17)	180 (17)
**TPOX**	231 (8)	231 (8)	231 (8)	231 (8)	231 (8)	231 (8)
**D5S818**	153 (11)	157 (12)	153 (11)	157 (12)	153 (11)	157 (12)
**Amelogenin**	108 (X)	108 (X)	108 (X)	108 (X)	108 (X)	108 (X)

B.

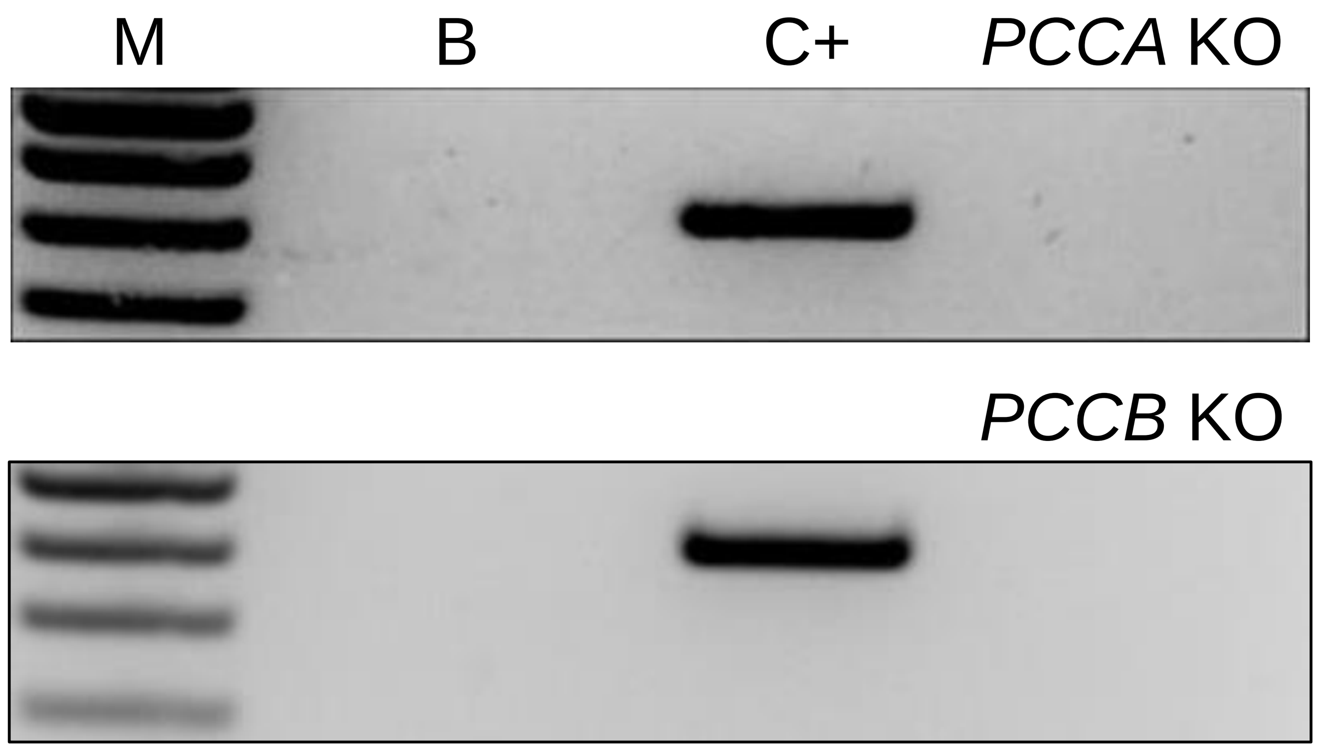

Supplement: Supplementary file 1 — (PDF 998 KB) [file 13577_2025_1193_MOESM1_ESM.pdf]
